# Diseases of the Stomach, Their Special Pathology, Diagnosis and Treatment

**Published:** 1902-10

**Authors:** 


Diseases of the Stomach, Their Special Pathology, Diagnosis and Treatment.—With sections on Anatomy, Physiology, Chemical and Miscroscopical Examination of Stomach Contents, Dietetics, Surgery of the Stomach, etc. By John C. Hemmeter, M. D., Philos D., Professor in the Medical Department in the University of Maryland, Baltimore; Consultant in the University Hospital and Director of the Clinical Laboratory; Author of "A Treatise on Diseases of the Intestines," etc., with many illustrations, a number of which are in colors and a lithograph frontispiece. Third enlarged and revised edition. Blakiston's Sons & Co., 1012 Walnut Street, Philadelphia.

In his preface to the first edition, Dr. Hemmeter thus stated the object he had in mind in preparing this work: "My chief effort has been to furnish the general practitioner with a work from which he can readily acquaint himself with all that has been done in this important branch of medicine, to fit himself to make examinations, to take advantage of new methods of diagnosis, and to treat this very difficult class of diseases rationally and successfully." With this end in view he has handled his subject systematically and concisely, considering in order the special anatomy and physiology of the digestive organs, then the therapeutics and materia medica of stomach diseases, and following these he carefully considers the symptomatology, diagnosis, prognosis, pathology and treatment of all of the various diseases that affect the stomach.

In the revision much new material has been added, two-thirds of the book being actually rewritten. No pains have been spared to bring it thoroughly abreast of the times.

Already this work has become a classic, which, together with "Diseases of the Intestines," by the same author (also published by Blakiston's Sons & Co.), should have a place in every well equipped medical library. R.

---

The following books will have early attention:

A Text-Book of Materia Medica, Therapeutics and Pharmacology.—By George F. Butler, M. D. 896 pages; illustrated. Philadelphia and London: W. B. Saunders & Co. Cloth, $4.00, net; sheep or half Morocco, $5.00, net.

The Treatment of Fractures.—By Charles L. Scudder, M. D. Octavo, 480 pages, with 645 original illustrations. Philadelphia and London: W. B. Saunders & Co. Polished buckram, $4.50, net; half Morocco, $5.00, net.

Atlas and Epitome of Traumatic Fractures and Dislocations.—By Prof. Dr. H. Helferich. With 216 colored illustrations on 64 lithographic plates, 190 text cuts, and 353 pages of text. Philadelphia and London: W. B. Saunders & Co. Cloth, $3.00, net.

Essentials of Diseases of the Ear.—By E. B. Gleason, M. D. 16mo., volume of 214 pages, with 114 illustrations. Philadelphia and London: W. B. Saunders & Co. Cloth, $1.00, net.

Essentials of Histology.—By Louis Leroy, M. D. 16mo., volume of 263 pages, with 92 beautiful illustrations. Philadelphia and London: W. B. Saunders & Co. Cloth, $1.00, net.

Hare's Practical Diagnosis.—By Hobart Amory Hare, M. D. 698 pages, illustrated with 236 engravings and 25 plates. Lea Brothers & Co., Philadelphia and New York.

